# Parental relationship satisfaction, symptoms of depression and anger/hostility, and the moderating role of perceived social support—a prospective cohort study in the light of the COVID-19 pandemic

**DOI:** 10.3389/fpsyg.2025.1470241

**Published:** 2025-05-13

**Authors:** Josephine Kümpfel, Victoria Weise, Judith T. Mack, Susan Garthus-Niegel

**Affiliations:** ^1^Institute and Policlinic of Occupational and Social Medicine, Faculty of Medicine of the Technische Universität Dresden, Dresden, Germany; ^2^Institute for Systems Medicine (ISM), Faculty of Medicine, Medical School Hamburg, Hamburg, Germany; ^3^Department of Childhood and Families, Norwegian Institute of Public Health, Oslo, Norway

**Keywords:** relationship satisfaction, perceived social support, parental depression, parental anger/hostility, COVID-19 pandemic, DREAM study

## Abstract

**Background:**

The challenging early parenthood time and the strains of the COVID-19 pandemic negatively affected parental mental health, whereas relationship satisfaction and perceived social support acted protective. Previous research focused on mothers, and little is known about mental health factors besides depression. This study aimed to investigate how relationship satisfaction and perceived social support affected symptoms of depression and anger/hostility in mothers and fathers in the light of the COVID-19 pandemic and whether perceived social support moderated these associations.

**Methods:**

*n* = 1,414 mothers and *n* = 885 fathers participating in the prospective cohort DREAM study completed questionnaires 14 months and 2 years postpartum. Hierarchical moderated regression analyses were conducted, controlling for the phase in the COVID-19 pandemic (before, during lockdown, during easements, afterwards) during which mental health was assessed.

**Results:**

Greater relationship satisfaction and perceived social support 14 months postpartum predicted fewer symptoms of depression and anger/hostility 2 years postpartum. Greater perceived social support moderated the association between relationship satisfaction and paternal symptoms of anger/hostility. Mental health symptoms were not significantly associated with the COVID-19 pandemic.

**Limitations:**

Limitations concern the limited representation of the post-pandemic group and the undifferentiated measurement of social support sources.

**Conclusion:**

The importance of relationship satisfaction and perceived social support for parental mental health is highlighted. Perceived social support can enhance the protective effect relationship satisfaction has on paternal symptoms of anger/hostility. Implementing corresponding prevention and education classes could improve parental mental health.

## 1 Introduction

Becoming a family is a major event in couples’ lives. While this is a joyful occasion for most parents, it is also accompanied by numerous changes. Many couples experience a decrease in relationship satisfaction in the transition to parenthood (Mack et al., 2023; Keizer and Schenk, 2012). Further, with the onset of the COVID-19 pandemic, an additional decrease was observed (Pietromonaco and Overall, 2022; Temiz and Elsharnouby, 2022; Ahuja and Khurana, 2021).

However, relationship satisfaction is a key factor in mental health. Studies have found that greater relationship satisfaction is associated with less symptoms of depression (Çankaya and Dikmen, 2022; Røsand et al., 2012). It has also been linked to less aggressive behavior, such as intimate partner violence (Mojahed et al., 2024; Stith et al., 2008).

This protective role of relationship satisfaction is especially important in the postpartum and early parenthood period, which can be a very stressful and challenging time (Asselmann et al., 2020), with many parents suffering from mental health symptoms (Parfitt and Ayers, 2014): Around 14% of mothers (Liu et al., 2021) and 8% of fathers (Cameron et al., 2016) experience symptoms of postpartum depression (PPD). Maternal and paternal symptoms of depression can continue for years after birth (Goodman, 2004; Vliegen et al., 2014; Dennis et al., 2022). Further, elevated levels of stress can increase the risk of aggressive behavior in a relationship (Shortt et al., 2013; Capaldi et al., 2012). Therefore, it can be assumed that elevated stress experienced during this time can result in an increase in parental aggressiveness and anger, too. However, research on this is scarce.

Impaired parental mental health during the postpartum period has been shown to negatively affect several parental and child-related outcomes, e.g., the child’s social–emotional and cognitive development and mother-to-infant bonding (Junge et al., 2017; Polte et al., 2019; Slomian et al., 2019). While most studies focus on mothers, poor paternal mental health, such as depression and aggressive behavior, can also have adverse effects on the child’s development and increases the risk of the child developing behavioral difficulties (Garthus-Niegel and Kittel-Schneider, 2023; Fletcher et al., 2011; Clarke et al., 2007).

Good parental mental health becomes even more important considering how the COVID-19 pandemic has affected the global population’s mental health. It has led to an increased prevalence of psychiatric disorders and mental health symptoms, like depression and anger (Chen et al., 2021; Hossain et al., 2020). Symptoms of PPD were more severe in parents during the COVID-19 pandemic than in parents before the pandemic (Pinto and Figueiredo, 2023; Chmielewska et al., 2021). However, previous findings suggest that this association varies depending on the phase in the pandemic. A review conducted by Richter et al. (2021) found an increase in mental health symptoms during the first lockdown compared to the pre-pandemic situation and a decrease after lockdown restrictions eased. However, mental health levels did not reach the pre-pandemic levels after easements, suggesting that different phases in the COVID-19 pandemic may have affected mental health differently.

Given this vulnerability, it is important to better understand which factors beneficially affect the mental health of parents of young children. In addition to relationship satisfaction, social support has been linked to better mental health during the perinatal period (Biaggi et al., 2016; Asselmann et al., 2020). Greater perceived support, i.e., the availability of support when needed as perceived by an individual (Sarason et al., 1990), has been linked to positive mental health outcomes, e.g., less symptoms of depression (Wang et al., 2018). This association has also been shown for parents of young children, indicating a buffering effect of perceived social support (Hughes et al., 2020). The protective role of physical and social engagement during isolation was also emphasized in other populations (da Cruz et al., 2022), reinforcing the multifactorial nature of resilience during the COVID-19 pandemic.

Becoming a parent, however, is associated with changes in a person’s social network and therefore the availability of social support. While parents often need additional support to meet the challenges of childcare, they also lack the energy to keep in touch with friends. Rözer et al. (2017) found an increase in contact with family and neighbors but a decrease in contact with friends. Also, greater social support has been linked to better mental health during the COVID-19 pandemic (Szkody et al., 2021). Oppermann et al. (2021) found that social support was protective against parental stress and eliminated the negative association between stress and negative parenting behavior during lockdown. However, the amount of social support experienced by women in the perinatal period during the COVID-19 pandemic was lower than that experienced by women before the pandemic.

To the best of our knowledge, both the association between perceived social support and parental anger and how their interaction influences fathers during the early parenthood period has not been investigated yet. Furthermore, as social support and relationship satisfaction have individually been associated with less symptoms of depression, more information is needed on whether perceived social support acts as a moderator and whether it can weaken or strengthen the effect of relationship satisfaction on mental health.

Taken together, while much research has been conducted on the association of relationship satisfaction, social support, and parental symptoms of depression, more research is needed regarding the fathers’ perspective, the effect of relationship satisfaction and perceived social support on parental symptoms of anger, and how all those factors interacted in the special situation of the COVID-19 pandemic. Therefore, the current study aimed to investigate how relationship satisfaction and perceived social support affect parental symptoms of depression and anger during the first 2 years postpartum and in the light of the different phases in the COVID-19 pandemic. Further, it aimed to investigate whether perceived social support moderates the association between relationship satisfaction and mental health.

## 2 Methods

### 2.1 Design

This study is based on data drawn from the prospective multi-method cohort study Dresden Study on Parenting, Work, and Mental Health (DResdner Studie zu Elternschaft, Arbeit und Mentaler Gesundheit, DREAM). The DREAM study investigates parental work participation, role distribution, stress factors, and their influence on mental and physical health of parents and their children from pregnancy up to 4.5 years postpartum. The main questionnaire-based study currently consists of six measurement points, one during pregnancy (T1) and five postpartum (T2–T6). For the present study, only data from T1, T3 (14 months postpartum), and T4 (2 years postpartum) were used. For more details on the prospective longitudinal cohort DREAM study, see Kress et al., 2019.

The study was reviewed and approved by the Ethics Committee of the Technische Universität Dresden (No: EK 278062015). All participants provided written informed consent to participate in this study.

### 2.2 Sample

The study comprises a community sample of mothers and their partners from Dresden, Germany. Participants were recruited during pregnancy, mainly at birth information evenings in obstetric clinics and in midwife practices from 2017 until the end of 2020. The participation was voluntary. Participants did not receive any financial compensation, although they were given small incentives.

For the current analyses, participants who did not meet the following criteria were excluded: not having completed the T3 and T4 questionnaires in time (i.e., within ±2 months of the questionnaire being due), not being in a heterosexual relationship with the same partner at T3 and T4, living in separate households from their partner at T3 or T4, or due to missing data in relevant questionnaires. After applying strict inclusion criteria, the final sample consisted of 2,299 participants: 1,414 mothers and 885 fathers.

### 2.3 Measures

Relationship satisfaction was assessed at T3 with the German short version of the Partnership Questionnaire (PFB-K; Kliem et al., 2012). It consists of nine items, e.g., “He or she tells me that he or she cares about me” which are rated on a 4-point scale ranging from “never/very rarely” (0) to “very often” (3). The sum score ranges from 0 to 27, with a higher score indicating higher relationship satisfaction. In this sample, the reliability of the PFB-K scale was high for mothers (α = 0.83) and fathers (α = 0.82).

Symptoms of depression during the past week were assessed at T4 with the 10-item Edinburgh Postnatal Depression Scale (EPDS; Cox et al., 1987; Bergant et al., 1998). Each item, e.g., “I have looked forward with enjoyment to things” was answered on a 4-point scale ranging from strongly disagreeing (e.g., “no, not at all,” 0) to strongly agreeing (e.g., “yes, most of the time,” 3). A higher total score (0–30) indicates greater severity of symptoms of depression (≥10 for mild and ≥13 for moderate to severe clinically relevant symptoms of depression). Reliability for mothers (α = 0.86) and fathers (α = 0.84) was high.

Symptoms of anger/hostility during the last week were assessed at T4 with the anger/hostility subscale of the German version of the Symptom Checklist (SCL-90-R; Franke, 2002). Each of the six items, such as how much they were bothered by having urges to beat, injure, or harm someone, was rated on a 5-point scale reaching from “not at all” (0) to “extremely” (4). Higher scores (0–24) indicate greater levels of anger/hostility. The present study yielded high reliabilities for mothers (α = 0.80) and fathers (α = 0.81).

Perceived social support was assessed at T3 with the German short version of the Social Support Questionnaire (F-SozU K-14; Fydrich et al., 2009). It consists of 14 items, e.g., “I have a group of people that I belong to and that I meet up with often.” Participants were asked to rate each item on a 5-point scale ranging from “totally disagree” (1) to “totally agree” (5), which was calculated into a mean score, ranging from 1 to 5, with a higher score indicating a greater level of perceived social support. The reliability in this sample was excellent for mothers (α = 0.92) and fathers (α = 0.93). The questionnaire has been implemented and shown reliable in other studies during the COVID-19 pandemic (e.g., Rösel et al., 2022).

Current phase in the COVID-19 pandemic at T4, at which the outcome variables were measured (i.e., symptoms of depression and anger/hostility), was assessed as a confounder. As it was of special interest for the research question, whether the phase in the pandemic was associated with the results, this confounder was included in all analyses. Participants were assigned to one of four groups, depending on whether they filled out the questionnaire (a) before the outbreak of the pandemic (i.e., before March 10, 2020), (b) during a lockdown/time of severe restrictions, (c) during a time of easements, or (d) after the pandemic ended (i.e., after January 16, 2023). The dates were chosen according to the pandemic situation in Dresden, Germany: On March 10, 2020, first restrictions were imposed, while on January 16, 2023, people were not required to wear face masks in public transport anymore, indicating a great loosening of restrictions. For the main analyses, dummy variables were used with the group “before the outbreak of the pandemic” as reference group.

Further confounders assumed to be associated with the outcomes based on previous research were selected. These include number of children (using data from T1–T3), academic degree at T1, employment at T3, and the use of facility-based childcare (daycare or childminder) at T4. Parents with multiples were considered using facility-based childcare if all their children were enrolled.

### 2.4 Data analysis

All analyses were conducted with IBM SPSS Statistics (Versions 27 and 29) using the SPSS modeling tool PROCESS (Hayes, 2022) and performed separately for mothers and fathers. Sex differences were compared descriptively. Data were checked for univariate outliers by means of box plots and excluded if they deviated more than ±3.0 *SD* from the mean. Multivariate outliers using the Mahalanobis distance were checked. If there were multivariate outliers, sensitivity analyses with outliers excluded were conducted. Attrition analyses were performed comparing those who completed the questionnaires PFB-K, EPDS, SCL-90-R, and F-SozU K-14 until T4 and those who did not. Descriptive analyses were performed for sociodemographic characteristics and all study variables. Test prerequisites for moderated regression analyses were checked. All analyses were conducted once without and once with the confounders that were significantly associated with the outcomes. For comparability reasons, the analyses for mothers and fathers were performed with the same set of confounders.

Hierarchical regression analyses consisting of four models were performed to investigate the association of relationship satisfaction with symptoms of depression and anger/hostility, and to examine the moderating role of social support. In the first model, only confounders were included. As the phase in the COVID-19 pandemic was dummy coded, the categories of having filled out the questionnaires on the outcomes during lockdown, during a time of easements and after the pandemic were included in the model. For the second and third model, the predictors relationship satisfaction and perceived social support were added. In the fourth model, the interaction term of relationship satisfaction and perceived social support was added. Where the interaction term became significant, follow-up analyses (i.e., simple slopes) were conducted using PROCESS (Hayes, 2022). The level of significance of *p* < 0.05 with a 95% bias corrected and accelerated confidence interval (BCa CI) based on 2,000 bootstrap samples was used. In some cases, only 1,999 iterations were possible, since the dummy variable of having filled out the questionnaires after the pandemic comprised a very small part of the sample (*n* = 8 mothers and fathers, respectively). In these cases, percentile confidence intervals are reported. Since all models comprise several predictors, adjusted *R^2^*s are reported. Sensitivity analyses with outliers excluded were conducted. In cases where the results of the sensitivity analyses regarding the final model differed from the regular analyses, they were reported as well (full results of the sensitivity analyses can be found in the Supplementary material).

## 3 Results

### 3.1 Descriptive statistics

Sample characteristics of the *n* = 2,299 parents (61.5% mothers, 38.5% fathers) are shown in Table 1. On average, participants had been in their relationship for 8 years. Regarding the COVID-19 pandemic, most participants completed the T4 questionnaires on the outcome variables during a time of easements (mothers: 71.9%, fathers: 70.6%), while fewer participants completed them before the pandemic or during a lockdown (mothers: 11.3%, 16.22%; fathers: 12.3%, 16.2%, respectively). Only some completed the questionnaires after the pandemic (mothers: 0.6%, fathers: 1.0%).

**Table 1:** Descriptive statistics.

Sample characteristics	Mothers (*n* = 1,414)^a^	Fathers (*n* = 885)^a^
Age^b^	30.28 ± 3.87 (18–43)	32.52 ± 5.01 (20–56)
Mother tongue^b^		
German	1,357 (95.97)	863 (97.51)
Other	57 (4.03)	22 (2.49)
Number of children		
One	1,133 (80.64)	691 (80.63)
Two	230 (16.37)	129 (15.05)
Three or more	42 (2.99)	37 (4.32)
Academic degree		
Academic degree	825 (58.55)	509 (58.64)
No academic degree	584 (41.45)	359 (41.36)
Employment		
Yes	766 (54.40)	793 (90.01)
No	642 (45.60)	88 (9.99)
Facility-based childcare		
Yes	1,324 (93.97)	821 (92.98)
No	85 (6.03)	62 (7.02)
Relationship duration (in years)^c^	8.24 ± 4.08 (1.28–27.40)	8.18 ± 3.90 (1.80–24.11)
Phase in the COVID-19 pandemic		
Before the pandemic	160 (11.33)	108 (12.26)
During lockdown	229 (16.22)	143 (16.23)
Time of easements	1,015 (71.88)	622 (70.60)
After the pandemic	8 (0.57)	8 (0.91)
PFB-K	18.92 ± 4.69 (3–27)	18.33 ± 4.51 (3–27)
F-SozU K-14	4.26 ± 0.64 (1–5)	4.07 ± 0.72 (1.07–5)
EPDS	6.36 ± 4.64 (0–25)	4.43 ± 4.01 (0–25)
Score < 10	1,100 (77.79)	779 (88.02)
Score ≥ 10 and < 13	161 (11.39)	69 (7.80)
Score ≥ 13	153 (10.82)	37 (4.18)
SCL-90-R	2.71 ± 3.02 (0–24)	1.94 ± 2.51 (0–17)

*n* (%) or *M* ± *SD* (range). PFB-K = Partnerschaftsfragebogen (partnership questionnaire), range 0 to 27; F-SozU K-14 = Fragebogen zur sozialen Unterstützung (perceived social support questionnaire), range 1–5; EPDS = Edinburgh Postnatal Depression Scale, range 0–30, scores ≥ 10 indicate clinically relevant symptoms of depression, scores ≥ 13 indicate moderate to severe symptoms of depression; SCL-90-R = anger/hostility subscale of the Symptom-Checklist-90-Revised, range 0 to 27. ^a^ *n* varied slightly due to missing data. ^b^ Assessed at T1. ^c^ Assessed at T3.

Compared to fathers, mothers reported significantly higher relationship satisfaction, *M*_mothers_ = 18.92, *M*_fathers_ = 18.33; *t*(1930,10) = 2.98, *p* = 0.005, and perceived social support, *M*_mothers_ = 4.26, *M*_fathers_ = 4.07; *t*(1725,54) = 6.29, *p* < 0.001. Mothers also reported more symptoms of depression in general, *M*_mothers_ = 6.36, *M*_fathers_ = 4.43; *t*(2076,82) = 10.55, *p* < 0.001, and more often clinically relevant and moderate to severe symptoms of depression than fathers, χ^2^(2) = 43.01, *p* < 0.001, Cramér’s V = 0.14. They further reported more symptoms of anger/hostility, *M*_mothers_ = 2.71, *M*_fathers_ = 1.94; *t*(2,123,67) = 6.59, *p* < 0.001, than fathers.

### 3.2 Correlation analyses

Correlation analyses examining associations between the predictor, outcomes, moderator, and potential confounders were performed with 2,000 bootstrapping iterations. For detailed results, see Tables 2, 3. Regarding the phase in the COVID-19 pandemic, only having completed the T4 questionnaires after the pandemic was significantly positively associated with the outcomes symptoms of depression, *r* = 0.085, *p* = 0.011, BCa 95% CI [−0.018, 0.193] and anger/hostility, *r* = 0.093, *p* = 0.006, [−0.032, 0.241] for fathers. However, for both effects, bias-corrected and accelerated bootstrap confidence intervals included 0. As the phase in the COVID-19 pandemic was of special interest for the research question, though, all of the phases except for the phase before the pandemic which served as the reference category for the dummy variables, were included in the further analyses for mothers and fathers.

**Table 2:** Correlation matrix including the predictor, moderator, outcomes, and potential confounders for mothers.

Variable	1	2	3	4	5	6	7	8	9.	10	11	12
1. PFB-K	–											
2. F-SozU K-14	0.326*** [0.280, 0.373]	–										
3. EPDS	−0.226*** [−0.280, −0.175]	−0.250*** [−0.300, −0.197]	–									
4. SCL-90-R	−0.200*** [−0.257, −0.146]	−0.207*** [−0.260, −0.154]	0.637*** [0.598, 0.677]	–								
5. Before the pandemic	0.032 [−0.024, 0.087]	−0.003 [−0.059, 0.049]	−0.037 [−0.082 0.013]	−0.007 [−0.056, 0.046]	–							
6. During lockdown	−0.026 [−0.080, 0.028]	0.007 [−0.046, 0.062]	−0.024 [−0.071, 0.025]	−0.044 [−0.086, 0.003]	0.156*** [−0.171, −0.140]	–						
7. During easements	0.006 [−0.049, 0.059]	0.003 [−0.048, 0.054]	0.038 [−0.010, 0.083]	0.033 [−0.015, 0.078]	0.573*** [−0.616, −0.530]	0.702*** [−0.741, −0.663]	–					
8. After the pandemic	−0.039 [−0.101, 0.013]	−0.042 [−0.124, 0.021]	0.047 [−0.032, 0.130]	0.045 [−0.015, 0.119]	−0.027 [−0.042, −0.010]	−0.033 [−0.052, −0.012]	−0.122*** [−0.171, −0.060]	–				
9. Number of children	−0.088** [−0.135, −0.041]	−0.069** [−0.124, −0.016]	0.020 [−0.030, 0.075]	0.054* [0.006, 0.107]	0.001 [−0.051, 0.058]	−0.042 [−0.085, 0.002]	0.030 [−0.027, 0.088]	0.022 [−0.033, 0.096]	–			
10. Academic degree	−0.036 [−0.087, 0.017]	0.038 [−0.018, 0.093]	−0.023 [−0.073, 0.026]	−0.031 [−0.080, 0.018]	−0.014 [−0.065, 0.037]	0.017 [−0.035, 0.069]	−0.005 [−0.056, 0.050]	0.006 [−0.056, 0.054]	0.003 [−0.049, 0.051]	–		
11. Employment	0.009 [−0.042, 0.062]	0.036 [−0.014, 0.084]	−0.012 [−0.065, 0.040]	0.001 [−0.051, 0.054]	0.042 [−0.011, 0.097]	−0.031 [−0.083, 0.021]	−0.007 [−0.061, 0.049]	0.012 [−0.043, 0.061]	−0.043 [−0.093, 0.007]	0.053* [−0.001, 0.109]	–	
12. Facility-based childcare	−0.020 [−0.071, 0.034]	−0.056* [−0.008, 0.123]	0.031 [−0.028, 0.091]	−0.012 [−0.078, 0.049]	−0.013 [−0.071, 0.041]	−0.028 [−0.087, 0.028]	0.035 [−0.018, 0.093]	−0.020 [−0.109, 0.024]	−0.069* [−0.151, 0.009]	0.042 [−0.012, 0.098]	0.195*** [0.145, 0.242]	–

Two-tailed testing. Confidence intervals are bias corrected and accelerated (BCa) and based on 2,000 bootstrap samples. *r* [BCa 95% CI]. PFB-K = Partnerschaftsfragebogen (partnership questionnaire); F-SozU K-14 = Fragebogen zur sozialen Unterstützung (perceived social support questionnaire); EPDS = Edinburgh Postnatal Depression Scale; SCL-90-R anger/hostility = anger/hostility subscale of the Symptom-Checklist-90-Revised. Significant results are in bold. *p < 0.05; **p < 0.01; ***p < 0.001.

**Table 3:** Correlation matrix including the predictor, moderator, outcomes, and potential confounders for fathers.

Variable	1.	2.	3.	4.	5.	6.	7.	8.	9.	10.	11.	12.
1. PFB-K	–											
2. F-SozU K-14	0.413*** [0.355, 0.475]	–										
3. EPDS	−0.244*** [−0.307, −0.178]	−0.282*** [−0.345, −0.216]	–									
4. SCL-90-R	−0.173*** [−0.240, −0.105]	−0.167*** [−0.231, −0.102]	0.603*** [0.538, 0.661]	–								
5. Before the pandemic	0.013 [−0.053, 0.078]	−0.010 [−0.077, 0.055]	−0.026 [−0.087, 0.036]	−0.037 [−0.089, 0.023]	–							
6. During lockdown	−0.011 [−0.072, 0.057]	−0.013 [−0.077, 0.052]	0.031 [−0.035, 0.094]	0.054 [−0.015, 0.129]	−0.163*** [−0.185, −0.142]	–						
7. During easements	0.005 [−0.059, 0.070]	0.019 [−0.047, 0.086]	−0.024 [−0.086, 0.044]	−0.037 [−0.104, 0.029]	−0.574*** [−0.623, −0.528]	−0.686*** [−0.734, −0.636]	–					
8. After the pandemic	−0.028 [−0.104, 0.041]	−0.008 [−0.069, 0.044]	0.085* [−0.018, 0.193]	0.093** [−0.032, 0.241]	−0.036 [−0.050, −0.022]	−0.043 [−0.059, −0.026]	−0.153*** [−0.204, −0.091]	–				
9. Number of children	−0.127*** [−0.205, −0.045]	−0.150*** [−0.218, −0.082]	0.100** [0.025, 0.173]	0.136*** [0.047, 0.231]	−0.015 [−0.073, 0.050]	0.025 [−0.040, 0.095]	−0.005 [−0.077, 0.066]	−0.021 [−0.050, 0.022]	–			
10. Academic degree	0.013 [−0.055, 0.085]	0.032 [−0.034, 0.100]	−0.009 [−0.078, 0.053]	−0.043 [−0.112, 0.027]	0.041 [−0.030, 0.111]	0.007 [−0.061, 0.077]	−0.041 [−0.114, 0.026]	0.031 [−0.043, 0.084]	−0.062 [−0.137, 0.011]	–		
11. Employment	0.022 [−0.045, 0.090]	−0.001 [−0.065, 0.065]	−0.016 [−0.095, 0.067]	−0.022 [−0.100, 0.051]	−0.010 [−0.081, 0.059]	0.009 [−0.064, 0.073]	0.018 [−0.052, 0.085]	−0.089* [−0.205, 0.026]	0.085* [0.021, 0.135]	−0.068 [−0.133, 0.003]	–	
12. Facility-based childcare	−0.027 [−0.106, 0.055]	0.006 [−0.068, 0.086]	−0.024 [−0.103, 0.050]	−0.034 [−0.114, 0.041]	0.022 [−0.054, 0.082]	−0.068* [−0.152, 0.011]	0.045 [−0.027, 0.114]	−0.024 [−0.134, 0.031]	0.036 [−0.024, 0.085]	−0.058 [−0.118, 0.005]	−0.008 [−0.064, 0.058]	–

Two-tailed testing. Confidence intervals are bias corrected and accelerated (BCa) and based on 2,000 bootstrap samples. *r* [BCa 95% CI]. PFB-K = Partnerschaftsfragebogen (partnership questionnaire); F-SozU K-14 = Fragebogen zur sozialen Unterstützung (perceived social support questionnaire); EPDS = Edinburgh Postnatal Depression Scale; SCL-90-R anger/hostility = anger/hostility subscale of the Symptom-Checklist-90-Revised. Significant results are in bold. *p < 0.05; **p < 0.01; ***p < 0.001.

For mothers and fathers, having more children was associated with lower relationship satisfaction and perceived social support as well as higher levels in symptoms of depression for fathers and anger/hostility for both. For mothers, having their child enrolled in facility-based childcare was significantly correlated with perceived social support, *r* = −0.056, *p* = 0.038, [−0.008, 0.123]. However, this association is questionable, as the confidence interval included 0. Therefore, as the potential confounders academic degree, employment, and facility-based childcare did not correlate with either of the outcomes in neither mothers nor fathers, they were excluded from further analyses.

### 3.3 Attrition analyses

Attrition analyses for sociodemographic and study variables were conducted. There were significant differences between completers and non-completers regarding mother tongue, academic degree, and phase in the COVID-19 pandemic.

Completers more often had German as their mother tongue, regarding mothers (96.0% vs. 92.8%; χ^2^(1) = 8.34, *p* = 0.004, φ = 0.07) and fathers (96.5% vs. 94.7%; χ^2^(1) = 7.45, *p* = 0.006, φ = 0.07). Completing mothers more often held an academic degree than non-completing mothers (58.6% vs. 48.8%), χ^2^(1) = 14.52, *p* < 0.001, φ = 0.09, as did fathers (58.6% vs. 40.7%), χ^2^(1) = 40.01, *p* < 0.001, φ = 0.17. Additionally, in mothers, completers more often filled out the T4 questionnaires before the pandemic or during lockdown, while non-completing mothers more often filled out the questionnaires during a time of easements or after the pandemic (Fisher–Freeman–Halton exact test, *p* = 0.038).

### 3.4 Regression and moderated analyses

Hierarchical regression analyses consisting of four models each were performed. The results of the final models including the confounders number of children and phase in the COVID-19 pandemic, the predictors relationship satisfaction and perceived social support, as well as their interaction term will be reported (with full details in the Supplementary material).

#### 3.4.1 Symptoms of depression

For maternal symptoms of depression, the first model explained 0.2% of variance, the second 5.2%, the third 8.5%, and the final model (Table 4) also explained 8.5% of variance in total. Relationship satisfaction (β = −0.165, *p* < 0.001, BCa 95% CI [−0.221, −0.107]) and perceived social support (β = −0.199, *p* < 0.001, [−1.894, −1.037]) were significant predictors of symptoms of depression. The interaction term did not become significant (β = −0.021, *p* = 0.437, [−0.109, 0.045]).

**Table 4:** Hierarchical regression of relationship satisfaction, perceived social support, and their interaction for symptoms of depression.

	*B*	*SE B*	β	BCa 95% CI		*p*	*R^2^_adj_*
				LL	UL		
Mothers							0.085
Constant	6.073	0.464		5.136	7.011	0.000	
Number of children	−0.106	0.238	−0.012	−0.554	0.357	0.658	
During lockdown	0.168	0.446	0.013	−0.662	1.010	0.696	
During easements	0.558	0.379	0.054	−0.181	1.297	0.138	
Post pandemic	2.461	2.394	0.040	−2.135	7.346	0.269	
PFB-K^a^	−0.164	0.028	**−0.165**	−0.221	−0.107	0.000	
F-SozU K-14^a^	−1.444	0.217	**−0.199**	−1.894	−1.037	0.000	
PFB-K^a^ x F-SozU K-14^a^	−0.032	0.041	−0.021	−0.109	0.045	0.437	
Fathers^b^							0.105
Constant	3.746	0.436		2.903	4.670	0.000	
Number of children	0.328	0.253	0.045	−0.167	0.803	0.188	
During lockdown	0.520	0.455	0.048	−0.399	1.414	0.248	
During easements	0.294	0.351	0.033	−0.446	1.010	0.403	
Post pandemic	3.716	2.268	0.089	−0.510^c^	8.592	0.084	
PFB-K^a^	−0.133	0.035	**−0.151**	−0.201	−0.063	0.000	
F-SozU K-14^a^	−1.271	0.234	**−0.228**	−1.741	−0.804	0.000	
PFB-K^a^ x F-SozU K-14^a^	−0.053	0.037	−0.049	−0.123	0.021	0.152	

BCa CI = bias corrected and accelerated confidence interval based on 2,000 bootstrap samples; LL = lower limit; UL = upper limit; PFB-K = Partnerschaftsfragebogen (partnership questionnaire); F-SozU K-14 = Fragebogen zur sozialen Unterstützung (perceived social support questionnaire). Significant results are in bold. ^a^ Mean-centered. ^b^ Based on 1,999 bootstrap samples. ^c^ Since a BCa CI could not be calculated in this case, the percentile bootstrap confidence interval is reported instead.

Regarding paternal symptoms of depression, the interaction term did not become significant either (β = −0.049, *p* = 0.152, [−0.123, 0.021]). Relationship satisfaction (β = −0.151, *p* < 0.001, [−0.201, −0.063]) and perceived social support (β = −0.228, *p* < 0.001, [−1.741, −0.804]) significantly predicted symptoms of depression. The first model accounted for 1.3% of variance, the second 6.6%, the third 10.4%, and the final model (Table 4) accounted for 10.5% of variance in total.

#### 3.4.2 Symptoms of anger/hostility

For mothers, the interaction term did not become significant (β = 0.015, *p* = 0.607, BCa 95% CI [−0.042, 0.069]), whereas relationship satisfaction (β = −0.148, *p* < 0.001, [−0.131, −0.060]) and perceived social support (β = −0.152, *p* < 0.001, [−1.009, −0.454]) were significantly associated with symptoms of anger/hostility. The first model explained 0.4% of variance, the second 4.2%, the third 6.3%, and the final model (Table 5) explained 6.2% of variance.

**Table 5:** Hierarchical regression of relationship satisfaction, perceived social support, and their interaction for symptoms of anger/hostility.

	*B*	*SE B*	β	BCa 95% CI		*p*	*R^2^_adj_*
				LL	UL		
Mothers							0.062
Constant	2.479	0.297		1.916	3.108	0.000	
Number of children	0.178	0.149	−0.030	−0.094	0.462	0.238	
During lockdown	−0.299	0.279	−0.036	−0.863	0.232	0.285	
During easements	0.055	0.255	0.008	−0.477	0.540	0.817	
Post pandemic	1.267	1.335	0.032	−0.890	3.537	0.324	
PFB-K^a^	−0.096	0.019	**−0.148**	−0.131	−0.060	0.000	
F-SozU K-14^a^	−0.716	0.143	**−0.152**	−1.009	−0.454	0.000	
PFB-K^a^ × F-SozU K-14^a^	0.014	0.028	0.015	−0.042	0.069	0.607	
Fathers^b^							0.060
Constant	1.258	0.324		0.548	1.930	0.000	
Number of children	0.451	0.218	**0.099**	0.064	0.855	0.036	
During lockdown	0.472	0.279	0.069	−0.078	1.013	0.089	
During easements	0.135	0.211	0.024	−0.283	0.538	0.524	
Post pandemic	2.583	2.061	0.099	−0.705	6.391	0.183	
PFB-K^a^	−0.065	0.022	**−0.118**	−0.110	−0.019	0.002	
F-SozU K-14^a^	−0.435	0.143	**−0.125**	−0.722	−0.170	0.005	
PFB-K^a^ × F-SozU K-14^a^	−0.056	0.022	**−0.083**	−0.098	−0.015	0.013	

BCa CI = bias corrected and accelerated confidence interval based on 2,000 bootstrap samples; LL = lower limit; UL = upper limit; PFB-K = Partnerschaftsfragebogen (partnership questionnaire); F-SozU K-14 = Fragebogen zur sozialen Unterstützung (perceived social support questionnaire). Significant results are in bold. ^a^ Mean-centered. ^b^ Based on 1,999 bootstrap samples.

Relationship satisfaction (β = −0.118, *p* = 0.002, [−0.110, −0.019]) and perceived social support (β = −0.125, *p* = 0.005, [−0.722, −0.170]) were significant predictors for paternal symptoms of anger/hostility, as well as the confounder number of children (β = 0.099, *p* = 0.036, [0.064, 0.855]). Also, the interaction term became significant (β = −0.083, *p* = 0.013, [−0.098, −0.015]). The first model covered 2.5% of variance, the second 4.7%, the third 5.4%, and the final model (Table 5) covered 6.0% of the total variance. The sensitivity analysis provided similar results. The interaction term became significant (β = −0.077, *p* = 0.017, [−0.079, −0.009]). Relationship satisfaction (β = −0.134, *p* = 0.001, [−0.102, −0.025]) and perceived social support (β = −0.130, *p* = 0.001, [−0.617, −0.152]) were significant predictors for paternal symptoms of anger/hostility, while the confounder number of children was not, which is different than in the analysis including outliers. The first model covered 1.0% of variance, the second 3.9%, the third 4.7%, and the final model covered 5.1% of variance.

### 3.5 Follow-up analysis

**Figure 1:**
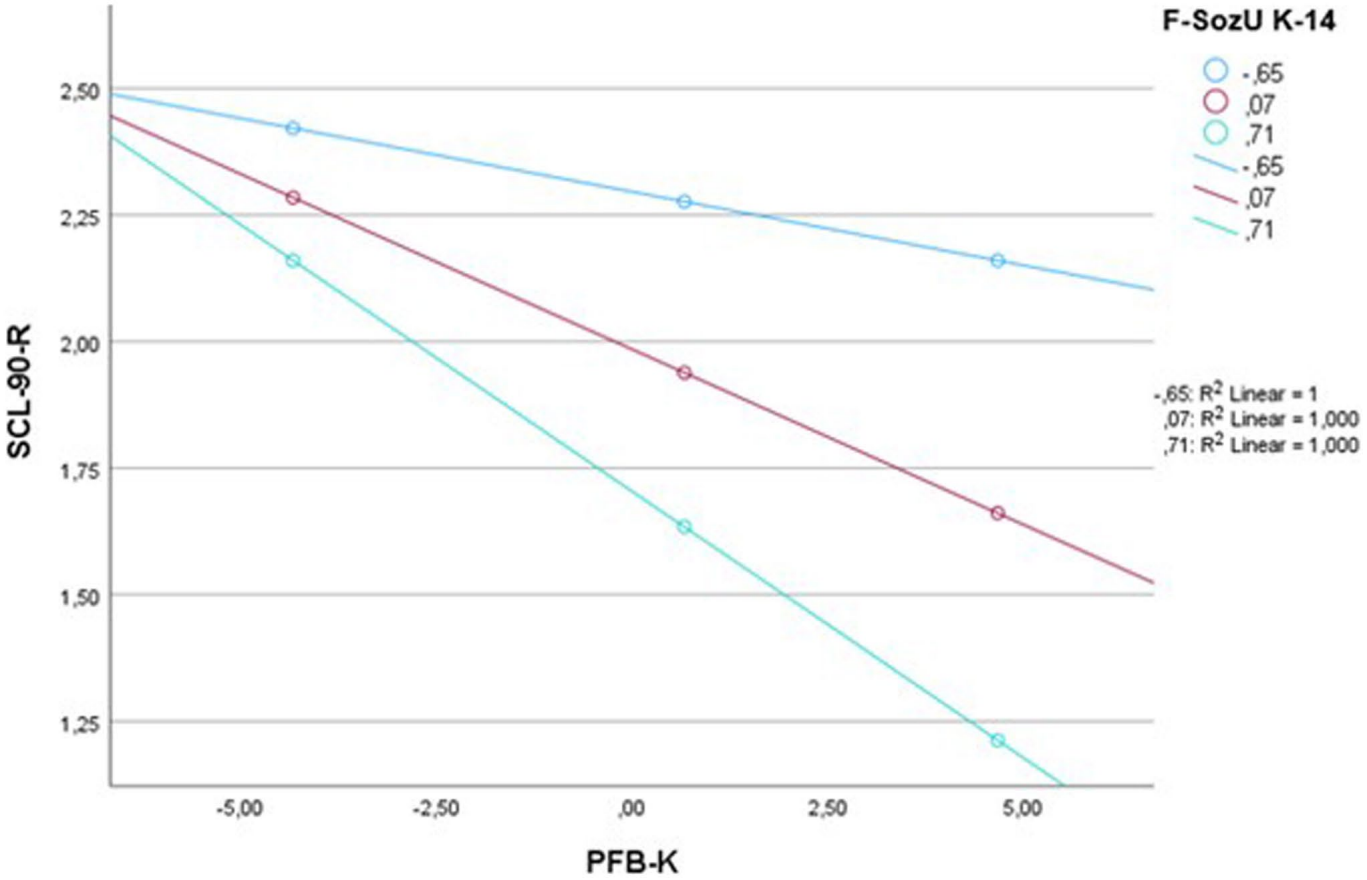
Simple slopes for the moderating effect of perceived social support on paternal symptoms of anger/hostility. SCL-90-R = anger/hostility subscale of the Symptom-Checklist-90-Revised; PFB-K = Partnerschaftsfragebogen (partnership questionnaire); F-SozU K-14 = Fragebogen zur sozialen Unterstützung (perceived social support questionnaire).

As the interaction term of relationship satisfaction and perceived social support became significant for paternal symptoms of anger/hostility, a follow-up analysis was conducted to further investigate the moderating role of perceived social support. Unstandardized simple slopes were conducted for perceived social support values at the 16th, 50th, and 84th percentile. Results are shown in Figure 1. The simple slope for the 16th percentile was B = −0.029 (95% CI [−0.077, 0.019]), *t* = −1.180, *p* = 0.238, for the 50th percentile B = −0.069 (95% CI [−0.109, −0.029]), *t* = −3.380, *p* = 0.001, and for the 84th percentile B = −0.105 (95% CI [−0.159, −0.052]), *t* = −3.847, *p* < 0.001. Thus, for higher values of perceived social support (i.e., the 50th and 84th percentile) there is a moderating effect on the association between relationship satisfaction and paternal symptoms of anger/hostility.

## 4 Discussion

This study aimed to replicate previous findings that relationship satisfaction and perceived social support act as protective factors regarding parental mental health (Çankaya and Dikmen, 2022; Røsand et al., 2012; Oppermann et al., 2021) and to provide new insights into how relationship satisfaction and perceived social support are associated with parental symptoms of anger/hostility. Further, it aimed to investigate whether perceived social support moderates the association between relationship satisfaction and mental health. As most previous studies focused on mothers, this large prospective cohort study aimed to also include the fathers’ perspective.

### 4.1 The association of relationship satisfaction and perceived social support with parental mental health

Relationship satisfaction as well as perceived social support at 14 months postpartum significantly predicted maternal and paternal mental health symptoms at 2 years postpartum, with greater relationship satisfaction and perceived social support being associated with less symptoms of depression and anger/hostility. This is in line with previous research and provides new insights into the importance of relationship satisfaction and perceived social support for parental symptoms of anger/hostility. The importance of relationship satisfaction is especially highlighted when considering the high prevalence of parental mental health symptoms: in this community sample, 22.2% of mothers and 12% of fathers reported clinically relevant or moderate to severe symptoms of depression at 2 years postpartum, supporting previous findings that symptoms of PPD can continue well into the second year after childbirth and beyond (Goodman, 2004; Vliegen et al., 2014; Dennis et al., 2022). Further, mothers reported significantly higher scores in symptoms of depression and anger/hostility than fathers. While research on differences in symptoms of anger/hostility in parents is scarce, findings regarding higher scores in symptoms of depression in mothers are in line with previous research, indicating that 14% of mothers (Liu et al., 2021) and 8% of fathers (Cameron et al., 2016) experience symptoms of depression. One possible explanation for this could be that after birth, women often function as the primary caretaker, which often involves more time pressure (Ruppanner et al., 2018). Other studies found that women experience pressure to meet parenting expectations as well as guilt for not meeting those expectations (Henderson et al., 2016). Thus, women could be more vulnerable to stress and impairments in mental health after becoming mothers. Also, mothers reported significantly higher scores in perceived social support and relationship satisfaction than fathers. Previous literature discussed that men and women differ in the way they participate in social relationships. Fuhrer et al. (1999) found that women have closer relationships and receive more social support than men, which could increase their perceived social support. A study conducted by Matud et al. (2003) discusses that gender differences regarding perceived social support could derive from socialization experiences, possibly leading to differences in social roles and behavior. Regarding gender differences in relationship satisfaction, previous studies found differing results. Bühler et al. (2021) discusses that while some studies found women reporting less relationship satisfaction than men, others indicate a decrease over time for men and still others find no gender differences regarding relationship satisfaction. Therefore, we think that our study contributes interesting new data to this question.

Surprisingly, findings that the COVID-19 pandemic would negatively affect parental mental health (Pinto and Figueiredo, 2023; Chmielewska et al., 2021) could not be replicated. Only having filled out the mental health questionnaires after the end of the pandemic was significantly associated with symptoms of depression and anger/hostility for fathers, which is in line with the finding that even though mental health improved again after restrictions eased, it did not fully return to a pre-pandemic level (Richter et al., 2021). However, as the confidence intervals included 0 and none of the phases during the pandemic became significant in the analyses, this finding should be considered with caution. A possible explanation for why none of the other phases were associated with parental mental health symptoms is that participants reported perceived social support scores above-average compared to the German population (Fydrich et al., 2009). Further, only participants who were living in the same household as their partner were included in the study. Participants of the current sample may have especially benefitted from the protective effects that social support and living with one’s intimate partner posed on mental health during the pandemic (Wirkner et al., 2021) and might have been less vulnerable to the otherwise often destabilizing effect of the COVID-19 pandemic.

Regarding a moderating role of perceived social support, an association was found for paternal symptoms of anger/hostility. Average and high levels of perceived social support significantly moderated the association between relationship satisfaction and symptoms of anger/hostility, indicating that the effect of relationship satisfaction on paternal symptoms of anger/hostility was stronger when perceived social support was greater. Thus, greater perceived support seems to enhance the protective effect relationship satisfaction has on paternal symptoms of anger/hostility. Furthermore, the results suggest that greater perceived social support could compensate for a less satisfactory relationship: When relationship satisfaction was low, fathers reporting greater perceived social support showed less symptoms of anger/hostility than fathers reporting less perceived social support. The moderation was not influenced by the phase in the COVID-19 pandemic, which again might be due to the protective role of the above-average levels of perceived social support in this sample and living with one’s partner. Further, although significant, the variance in paternal symptoms of anger/hostility explained through the investigated moderation remains modest (6.0% and 5.1% for the models including and excluding outliers, respectively). This might be due to the multifactorial nature of mental health (McNamara et al., 2021). Nevertheless, this finding provides valuable insights into a significant factor influencing paternal mental health, consequently opening up new possibilities on how to improve it.

Nonetheless, these findings highlight the importance of perceived social support for fathers who are less satisfied with their relationship. While satisfaction in romantic relationships is important for parental mental health (Çankaya and Dikmen, 2022; Røsand et al., 2012), these findings highlight the importance of perceived social support from other relationships as well. However, Umberson et al. (2022) found that young and middle-aged men are more at risk of social isolation than women. Thus, it becomes even more important to find ways of improving paternal experience of and access to social support.

This moderating effect was only found in one of four analyses, while there was a significant main effect of perceived social support on symptoms of depression and anger/hostility. Therefore, the findings of this study could suggest that perceived social support is linked to parental mental health not through a moderating association, but rather directly. As this is the first study to investigate this, more research is needed.

Mothers with more children reported lower relationship satisfaction, perceived social support, and more symptoms of anger/hostility, while fathers additionally reported more symptoms of depression, compared to parents with fewer children. In the regression analysis, a greater number of children significantly predicted more paternal symptoms of anger/hostility. This is in line with literature indicating that the birth of a second child increases the time pressure parents experience, which has consequences for parental mental health (Ruppanner et al., 2018).

### 4.2 Strengths and limitations

Several strengths of this study can be reported. It contributes to the growing body of research investigating not only maternal, but also paternal mental health and provides new insights into the association of relationship satisfaction and perceived social support with symptoms of depression and anger/hostility in the time of the COVID-19 pandemic. Much research regarding parental mental health is conducted in the first months postpartum, while this study investigates mental health 2 years postpartum. To the best of our knowledge, the question of whether perceived social support moderates the association between relationship satisfaction and parental symptoms of depression and anger/hostility has not been investigated yet, therefore this study introduces a new perspective on the relationship of those factors. Further, little is known about parental symptoms of anger/hostility, therefore, our study contributes new insights regarding this mental health aspect. Finally, the data for our study were drawn from a prospective multi-method cohort study, the DREAM study.

However, some limitations need to be considered. Attrition analyses showed that non-completing parents less often held a university degree and had German as their mother tongue than completing parents. In addition, the sample consisted only of participants living in the same household as their partner and participants reported above-average levels of perceived social support, which could pose a self-selection bias limiting the results’ generalizability. All measures used in this study are based on self-report questionnaires, which could lead to an overestimation or underestimation of the effects due to self-report bias. However, due to their practicality and ability to capture subjective perceptions in a large cohort study, we consider the application of self-report questionnaires in this study justified. Further, completing mothers more often reported on their mental health before the pandemic or during lockdown. As the completing sample reported above-average perceived social support, they might have been more resilient to the strains of lockdown than the general population, possibly resulting in an underestimation of the effect the pandemic had on maternal mental health in general. Also, results regarding the finding that having filled out the mental health questionnaires after the end of the pandemic significantly increased paternal symptoms of anger/hostility are based on a very small subsample (n = 8 per group). Thus, results concerning this phase lack statistical power and cannot be generalized. However, as the primary aim of this study was to investigate mental health during instead of after the pandemic, the sample can nevertheless be considered very suitable for answering the research question. Further, as mothers and fathers were treated as independent participants, data nesting within couples was not accounted for in this study. Finally, the perceived social support questionnaire used did not differentiate the sources of support (e.g., partner, family, or friends), limiting the derivation of specific recommendations regarding which source of support to focus on to enhance parental mental health.

### 4.3 Practical implications and future research

Our findings highlight the importance of relationship satisfaction and perceived social support for parental mental health. They also suggest a moderating association between perceived social support, relationship satisfaction, and paternal symptoms of anger/hostility, indicating that for fathers, greater perceived social support might compensate for a less satisfactory relationship. Since parental mental health is relevant for the whole family, improving it benefits society in a broader way.

Therefore, strengthening parental relationship satisfaction, perceived social support, and mental health as well as raising awareness for these factors is essential. Parenting education classes and couples interventions have been shown suitable measures (Pinquart and Teubert, 2010; Halford et al., 2018) and could be offered to expectant parents and parents in the postpartum time. Our findings further highlight that many fathers experience mental health symptoms in the early parenthood period and could therefore benefit from interventions that focus on strengthening relationship satisfaction and perceived social support. However, most interventions so far included only mothers (Pinquart and Teubert, 2010) but they should also address fathers in the future.

In addition to prevention, it may be beneficial for treating already developed mental health symptoms to focus more on strengthening the parental relationship and encouraging engagement in social activities. In treating PPD in mothers, such measures have already been established (Horowitz and Goodman, 2006; Alves et al., 2018) and should be increasingly extended to fathers.

Finally, in this study, none of the phases in the COVID-19 pandemic was associated with parental mental health symptoms 2 years postpartum. This could be due to participants reporting above-average levels of perceived social support. Therefore, enhancing perceived social support could increase resilience and help maintain parental mental wellbeing during times of crisis.

Being the first study, future research should further investigate the moderating role of perceived social support on the association between relationship satisfaction and mental health. Further, research should more frequently consider symptoms of anger/hostility for a broader understanding of parental mental health. Research should also continue to investigate the direct roles of relationship satisfaction and perceived social support for maternal and especially paternal mental health. Finally, future research should explore parental mental health after the end of the COVID-19 pandemic.

## 5 Conclusion

This study contributes to the literature on maternal and paternal mental health in the context of the COVID-19 pandemic, supporting previous findings and providing new insights into the beneficial association of relationship satisfaction and perceived social support with symptoms of depression and anger/hostility of mothers as well as fathers 2 years postpartum. Further, the results suggest a moderating role of perceived social support regarding paternal symptoms of anger/hostility. Finally, these findings highlight the importance of relationship satisfaction and perceived social support for treating and preventing maternal and paternal mental health symptoms.

## Data Availability

The datasets generated and/or analyzed during the current study are not publicly available due to legal and ethical constraints, as public sharing of participant data was not included in the informed consent of the study, but are available from the corresponding author on reasonable request. Requests to access the datasets should be directed to Susan Garthus-Niegel, susan.garthus-niegel@uniklinikum-dresden.de.
