# Supplementary material for: Parental relationship satisfaction, symptoms of depression and anger/hostility, and the moderating role of perceived social support—a prospective cohort study in the light of the COVID-19 pandemic
Source: Front Psychol. 2025 May 13;16:1470241. doi: 10.3389/fpsyg.2025.1470241 (PMC12106326; doi:10.3389/fpsyg.2025.1470241)
Supplement: Supplementary file 1 [file Supplementary_file_1.docx]

**Supplementary Material**

# Full Results of the Hierarchical Regression Analyses

**Table S.1**

*Hierarchical Regression Analysis of Relationship Satisfaction, Perceived Social Support, and Their Interaction for Maternal Symptoms of Depression, Controlled for Confounders*

|  | *B* | *SE B* | β | BCa 95% CI | | *p* | *R^2^_adj_* | *F for* Δ*R²* |
| --- | --- | --- | --- | --- | --- | --- | --- | --- |
|  |  |  |  | LL | UL |  |  |  |
| Model 1 |  |  |  |  |  |  | .002 | 1.662 |
| Constant | 5.643 | 0.475 |  | 4.707 | 6.614 | .000 |  |  |
| Number of children | 0.160 | 0.251 | .017 | -0.314 | 0.656 | .516 |  |  |
| During lockdown | 0.279 | 0.466 | .022 | -0.607 | 1.186 | .543 |  |  |
| During easement | 0.622 | 0.397 | .060 | -0.158 | 1.371 | .119 |  |  |
| Post pandemic | 3.387 | 2.690 | .055 | -1.961 | 8.960 | .168 |  |  |
| Model 2 |  |  |  |  |  |  | .052 | 16.338*** |
| Constant | 5.969 | 0.458 |  | 5.085 | 6.899 | .000 |  |  |
| Number of children | -0.018 | 0.244 | -.002 | -0.488 | 0.458 | .941 |  |  |
| During lockdown | 0.098 | 0.452 | .008 | -0.772 | 0.983 | .827 |  |  |
| During easement | 0.520 | 0.383 | .050 | -0.221 | 1.253 | .175 |  |  |
| Post pandemic | 2.761 | 2.614 | .045 | -2.369 | 8.023 | .248 |  |  |
| PFB-K^a^ | -0.225 | 0.027 | **-.226** | -0.267 | -0.173 | .000 |  |  |
| Model 3 |  |  |  |  |  |  | .085 | 22.748*** |
| Constant | 6.038 | 0.459 |  | 5.107 | 7.006 | .000 |  |  |
| Number of children | -0.095 | 0.238 | -.010 | -0.528 | 0.363 | .684 |  |  |
| During lockdown | 0.152 | 0.448 | .012 | -0.697 | 1.031 | .726 |  |  |
| During easement | 0.548 | 0.380 | .053 | -0.208 | 1.305 | .147 |  |  |
| Post pandemic | 2.444 | 2.382 | .040 | -2.121 | 7.263 | .270 |  |  |
| PFB-K^a^ | -0.163 | 0.028 | **-.164** | -0.221 | -0.105 | .000 |  |  |
| F-SozU K-14^a^ | -1.412 | 0.215 | **-.195** | -1.865 | -0.983 | .000 |  |  |
| Model 4 |  |  |  |  |  |  | .085 | 19.588*** |
| Constant | 6.073 | 0.464 |  | 5.136 | 7.011 | .000 |  |  |
| Number of children | -0.106 | 0.238 | -.012 | -0.554 | 0.357 | .658 |  |  |
| During lockdown | 0.168 | 0.446 | .013 | -0.662 | 1.010 | .696 |  |  |
| During easement | 0.558 | 0.379 | .054 | -0.181 | 1.297 | .138 |  |  |
| Post pandemic | 2.461 | 2.394 | .040 | -2.135 | 7.346 | .269 |  |  |
| PFB-K^a^ | -0.164 | 0.028 | **-.165** | -0.221 | -0.107 | .000 |  |  |
| F-SozU K-14^a^ | -1.444 | 0.217 | **-.199** | -1.894 | -1.037 | .000 |  |  |
| PFB-K^a^ x F-SozU K-14^a^ | -0.032 | 0.041 | -.021 | -0.109 | 0.045 | .437 |  |  |

*Note.* BCa CI = bias corrected and accelerated confidence interval based on 2,000 bootstrap samples; LL = lower limit; UL = upper limit; PFB-K = Partnerschaftsfragebogen (partnership questionnaire); F-SozU K-14 = Fragebogen zur sozialen Unterstützung (perceived social support questionnaire). Significant results are in bold.
^a^ Mean-centered.
**p* < .05. ***p* < .01. ****p* < .001.

**Table S.2**

*Hierarchical Regression Analysis of Relationship Satisfaction, Perceived Social Support, and Their Interaction for Maternal Symptoms of Anger/Hostility, Controlled for Confounders*

|  | *B* | *SE B* | β | BCa 95% CI | | *p* | *R^2^_adj_* | *F for* Δ*R²* |
| --- | --- | --- | --- | --- | --- | --- | --- | --- |
|  |  |  |  | LL | UL |  |  |  |
| Model 1 |  |  |  |  |  |  | .004 | 2.277 |
| Constant | 2.273 | 0.314 |  | 1.696 | 2.879 | .000 |  |  |
| Number of children | 0.314 | 0.158 | **.053** | 0.022 | 0.635 | .047 |  |  |
| During lockdown | -0.217 | 0.289 | -.026 | -0.817 | 0.373 | .442 |  |  |
| During easement | 0.102 | 0.260 | .015 | -0.439 | 0.636 | .704 |  |  |
| Post pandemic | 1.795 | 1.383 | .045 | -0.583 | 4.551 | .151 |  |  |
| Model 2 |  |  |  |  |  |  | .042 | 13.224*** |
| Constant | 2.459 | 0.311 |  | 1.887 | 3.059 | .000 |  |  |
| Number of children | 0.213 | 0.156 | .036 | -0.079 | 0.533 | .166 |  |  |
| During lockdown | -0.320 | 0.284 | -.039 | -0.907 | 0.246 | .259 |  |  |
| During easement | 0.044 | 0.257 | .007 | -0.486 | 0.579 | .865 |  |  |
| Post pandemic | 1.439 | 1.354 | .036 | -0.992 | 4.241 | .243 |  |  |
| PFB-K^a^ | -0.128 | 0.019 | **-.198** | -0.168 | -0.089 | .000 |  |  |
| Model 3 |  |  |  |  |  |  | .063 | 16.591*** |
| Constant | 2.494 | 0.298 |  | 1.950 | 3.113 | .000 |  |  |
| Number of children | 0.173 | 0.149 | .029 | -0.100 | 0.464 | .253 |  |  |
| During lockdown | -0.292 | 0.278 | -.036 | -0.855 | 0.238 | .290 |  |  |
| During easement | 0.059 | 0.255 | .009 | -0.471 | 0.542 | .808 |  |  |
| Post pandemic | 1.275 | 1.343 | .032 | -0.912 | 3.606 | .320 |  |  |
| PFB-K^a^ | -0.096 | 0.019 | **-.148** | -0.133 | -0.059 | .000 |  |  |
| F-SozU K-14^a^ | -0.730 | 0.139 | **-.155** | -1.005 | -0.467 | .000 |  |  |
| Model 4 |  |  |  |  |  |  | .062 | 14.257*** |
| Constant | 2.479 | 0.297 |  | 1.916 | 3.108 | .000 |  |  |
| Number of children | 0.178 | 0.149 | -.030 | -0.094 | 0.462 | .238 |  |  |
| During lockdown | -0.299 | 0.279 | -.036 | -0.863 | 0.232 | .285 |  |  |
| During easement | 0.055 | 0.255 | .008 | -0.477 | 0.540 | .817 |  |  |
| Post pandemic | 1.267 | 1.335 | .032 | -0.890 | 3.537 | .324 |  |  |
| PFB-K^a^ | -0.096 | 0.019 | **-.148** | -0.131 | -0.060 | .000 |  |  |
| F-SozU K-14^a^ | -0.716 | 0.143 | **-.152** | -1.009 | -0.454 | .000 |  |  |
| PFB-K^a^ x F-SozU K-14^a^ | 0.014 | 0.028 | .015 | -0.042 | 0.069 | .607 |  |  |

*Note.* BCa CI = bias corrected and accelerated confidence interval based on 2,000 bootstrap samples; LL = lower limit; UL = upper limit; PFB-K = Partnerschaftsfragebogen (partnership questionnaire); F-SozU K-14 = Fragebogen zur sozialen Unterstützung (perceived social support questionnaire). Significant results are in bold.
^a^ Mean-centered.
**p* < .05. ***p* < .01. ****p* < .001.

**Table S.3**

*Hierarchical Regression Analysis of Relationship Satisfaction, Perceived Social Support, and Their Interaction for Paternal Symptoms of Depression, Controlled for Confounders*

|  | *B* | *SE B* | β | BCa 95% CI | | *p* | *R^2^_adj_* | *F for* Δ*R²* |
| --- | --- | --- | --- | --- | --- | --- | --- | --- |
|  |  |  |  | LL | UL |  |  |  |
| Model 1 |  |  |  |  |  |  | .013 | 3.908** |
| Constant | 3.226 | 0.451 |  | 2.286 | 4.107 | .000 |  |  |
| Number of children | 0.679 | 0.266 | **.093** | 0.147 | 1.186 | .008 |  |  |
| During lockdown | 0.602 | 0.473 | .055 | -0.342 | 1.532 | .206 |  |  |
| During easement | 0.299 | 0.367 | .034 | -0.432 | 1.065 | .420 |  |  |
| Post pandemic | 4.011 | 2.462 | .097 | -0.329 | 8.684 | .081 |  |  |
| Model 2 |  |  |  |  |  |  | .066 | 13.094*** |
| Constant | 3.514 | 0.423 |  | 2.665 | 4.321 | .000 |  |  |
| Number of children | 0.459 | 0.250 | .063 | -0.022 | 0.946 | .065 |  |  |
| During lockdown | 0.557 | 0.459 | .051 | -0.367 | 1.437 | .231 |  |  |
| During easement | 0.286 | 0.351 | .033 | -0.420 | 1.038 | .419 |  |  |
| Post pandemic | 3.695 | 2.251 | .089 | -0.361 | 7.946 | .079 |  |  |
| PFB-K^a^ | -0.206 | 0.030 | **-.234** | -0.268 | -0.147 | .000 |  |  |
| Model 3^b^ |  |  |  |  |  |  | .104 | 17.396*** |
| Constant | 3.650 | 0.428 |  | 2.806 | 4.531 | .000 |  |  |
| Number of children | 0.322 | 0.254 | .044 | -0.175 | 0.794 | .201 |  |  |
| During lockdown | 0.583 | 0.451 | .054 | -0.311 | 1.463 | .191 |  |  |
| During easement | 0.325 | 0.347 | .037 | -0.396 | 1.013 | .348 |  |  |
| Post pandemic | 3.749 | 2.241 | .090 | -0.466^c^ | 8.583 | .076 |  |  |
| PFB-K^a^ | -0.130 | 0.035 | **-.147** | -0.200 | -0.060 | .000 |  |  |
| F-SozU K-14^a^ | -1.198 | 0.223 | **-.215** | -1.630 | -0.768 | .000 |  |  |
| Model 4^b^ |  |  |  |  |  |  | .105 | 15.218*** |
| Constant | 3.746 | 0.436 |  | 2.903 | 4.670 | .000 |  |  |
| Number of children | 0.328 | 0.253 | .045 | -0.167 | 0.803 | .188 |  |  |
| During lockdown | 0.520 | 0.455 | .048 | -0.399 | 1.414 | .248 |  |  |
| During easement | 0.294 | 0.351 | .033 | -0.446 | 1.010 | .403 |  |  |
| Post pandemic | 3.716 | 2.268 | .089 | -0.510^c^ | 8.592 | .084 |  |  |
| PFB-K^a^ | -0.133 | 0.035 | **-.151** | -0.201 | -0.063 | .000 |  |  |
| F-SozU K-14^a^ | -1.271 | 0.234 | **-.228** | -1.741 | -0.804 | .000 |  |  |
| PFB-K^a^ x F-SozU K-14^a^ | -0.053 | 0.037 | -.049 | -0.123 | 0.021 | .152 |  |  |

*Note.* BCa CI = bias corrected and accelerated confidence interval based on 2,000 bootstrap samples; LL = lower limit; UL = upper limit; PFB-K = Partnerschaftsfragebogen (partnership questionnaire); F-SozU K-14 = Fragebogen zur sozialen Unterstützung (perceived social support questionnaire). Significant results are in bold.
^a^ Mean-centered.
^b^ Based on 1999 bootstrap samples.
^c^ Since a BCa CI could not be calculated in this case, the percentile bootstrap confidence interval is reported instead.
**p* < .05. ***p* < .01. ****p* < .001.

**Table S.4**

*Hierarchical Regression Analysis of Relationship Satisfaction, Perceived Social Support, and Their Interaction for Paternal Symptoms of Anger/Hostility, Controlled for Confounders*

|  | *B* | *SE B* | β | BCa 95% CI | | *p* | *R^2^_adj_* | *F for* Δ*R²* |
| --- | --- | --- | --- | --- | --- | --- | --- | --- |
|  |  |  |  | LL | UL |  |  |  |
| Model 1 |  |  |  |  |  |  | .025 | 6.415*** |
| Constant | 0.997 | 0.323 |  | 0.312 | 1.634 | .005 |  |  |
| Number of children | 0.575 | 0.215 | **.126** | 0.195 | 1.003 | .007 |  |  |
| During lockdown | 0.550 | 0.296 | .081 | -0.020 | 1.122 | .064 |  |  |
| During easement | 0.162 | 0.219 | .029 | -0.267 | 0.580 | .456 |  |  |
| Post pandemic | 2.731 | 2.092 | .105 | -0.686 | 6.753 | .165 |  |  |
| Model 2 |  |  |  |  |  |  | .047 | 9.377*** |
| Constant | 1.115 | 0.323 |  | 0.436 | 1.731 | .001 |  |  |
| Number of children | 0.485 | 0.217 | **.107** | 0.111 | 0.930 | .029 |  |  |
| During lockdown | 0.532 | 0.291 | .078 | -0.038 | 1.094 | .072 |  |  |
| During easement | 0.156 | 0.218 | .028 | -0.256 | 0.564 | .482 |  |  |
| Post pandemic | 2.601 | 2.000 | .100 | -0.705 | 6.460 | .170 |  |  |
| PFB-K^a^ | -0.085 | 0.019 | **-.153** | -0.122 | -0.045 | .000 |  |  |
| Model 3^b^ |  |  |  |  |  |  | .054 | 9.178*** |
| Constant | 1.156 | 0.322 |  | 0.484 | 1.815 | .000 |  |  |
| Number of children | 0.444 | 0.218 | **.098** | 0.058 | 0.850 | .039 |  |  |
| During lockdown | 0.539 | 0.280 | .079 | -0.009 | 1.069 | .057 |  |  |
| During easement | 0.168 | 0.215 | .031 | -0.264 | 0.587 | .440 |  |  |
| Post pandemic | 2.617 | 2.035 | .101 | -0.625 | 6.324 | .171 |  |  |
| PFB-K^a^ | -0.062 | 0.022 | **-.112** | -0.107 | -0.014 | .007 |  |  |
| F-SozU K-14^a^ | -0.358 | 0.137 | **-.103** | -0.628 | -0.104 | .012 |  |  |
| Model 4^b^ |  |  |  |  |  |  | .060 | 8.702*** |
| Constant | 1.258 | 0.324 |  | 0.548 | 1.930 | .000 |  |  |
| Number of children | 0.451 | 0.218 | **.099** | 0.064 | 0.855 | .036 |  |  |
| During lockdown | 0.472 | 0.279 | .069 | -0.078 | 1.013 | .089 |  |  |
| During easement | 0.135 | 0.211 | .024 | -0.283 | 0.538 | .524 |  |  |
| Post pandemic | 2.583 | 2.061 | .099 | -0.705 | 6.391 | .183 |  |  |
| PFB-K^a^ | -0.065 | 0.022 | **-.118** | -0.110 | -0.019 | .002 |  |  |
| F-SozU K-14^a^ | -0.435 | 0.143 | **-.125** | -0.722 | -0.170 | .005 |  |  |
| PFB-K^a^ x F-SozU K-14^a^ | -0.056 | 0.022 | **-.083** | -0.098 | -0.015 | .013 |  |  |

*Note.* BCa CI = bias corrected and accelerated confidence interval based on 2,000 bootstrap samples; LL = lower limit; UL = upper limit; PFB-K = Partnerschaftsfragebogen (partnership questionnaire); F-SozU K-14 = Fragebogen zur sozialen Unterstützung (perceived social support questionnaire). Significant results are in bold.
^a^ Mean-centered.
^b^ Based on 1999 bootstrap samples.
**p* < .05. ***p* < .01. ****p* < .001.

# Full Results of the Sensitivity Analyses of the Hierarchical Regression Analyses Excluding Multivariate Outliers

**Table S.5**

*Hierarchical Regression Analysis of Relationship Satisfaction, Perceived Social Support, and Their Interaction for Maternal Symptoms of Depression, Controlled for Confounders and Excluding Multivariate Outliers*

|  | *B* | *SE B* | β | BCa 95% CI | | *p* | *R^2^_adj_* | *F for* Δ*R²* |
| --- | --- | --- | --- | --- | --- | --- | --- | --- |
|  |  |  |  | LL | UL |  |  |  |
| Model 1 |  |  |  |  |  |  | .002 | 1.854 |
| Constant | 5.255 | 0.457 |  | 4.376 | 6.136 | .000 |  |  |
| Number of children | 0.253 | 0.269 | .026 | -0.263 | 0.778 | .351 |  |  |
| During lockdown | 0.547 | 0.431 | .045 | -0.371 | 1.434 | .203 |  |  |
| During easement | 0.817 | 0.355 | **.080** | 0.089 | 1.595 | .029 |  |  |
| Model 2 |  |  |  |  |  |  | .054 | 20.419*** |
| Constant | 5.589 | 0.442 |  | 4.675 | 6.434 | .000 |  |  |
| Number of children | 0.099 | 0.260 | .010 | -0.393 | 0.618 | .702 |  |  |
| During lockdown | 0.341 | 0.420 | .028 | -0.534 | 1.151 | .417 |  |  |
| During easement | 0.664 | 0.343 | .065 | -0.044 | 1.392 | .057 |  |  |
| PFB-K^a^ | -0.225 | 0.026 | **-.230** | -0.277 | -0.175 | .000 |  |  |
| Model 3 |  |  |  |  |  |  | .087 | 27.134*** |
| Constant | 5.703 | 0.426 |  | 4.888 | 6.551 | .000 |  |  |
| Number of children | -0.007 | 0.250 | -.001 | -0.486 | 0.479 | .975 |  |  |
| During lockdown | 0.377 | 0.426 | .031 | -0.555 | 1.224 | .364 |  |  |
| During easement | 0.678 | 0.353 | .067 | -0.042 | 1.335 | .055 |  |  |
| PFB-K^a^ | -0.162 | 0.027 | **-.166** | -0.215 | -0.109 | .000 |  |  |
| F-SozU K-14^a^ | -1.398 | 0.206 | **-.195** | -1.825 | -1.005 | .000 |  |  |
| Model 4 |  |  |  |  |  |  | .087 | 22.669*** |
| Constant | 5.732 | 0.427 |  | 4.931 | 6.587 | .000 |  |  |
| Number of children | -0.017 | 0.250 | -.002 | -0.488 | 0.467 | .944 |  |  |
| During lockdown | 0.388 | 0.425 | .032 | -0.530 | 1.229 | .351 |  |  |
| During easement | 0.684 | 0.352 | .067 | -0.034 | 1.330 | .052 |  |  |
| PFB-K^a^ | -0.163 | 0.027 | **-.167** | -0.217 | -0.109 | .000 |  |  |
| F-SozU K-14^a^ | -1.424 | 0.208 | **-.199** | -1.849 | -1.034 | .000 |  |  |
| PFB-K^a^ x F-SozU K-14^a^ | -0.025 | 0.039 | -.017 | -0.099 | 0.054 | .519 |  |  |

*Note.* BCa CI = bias corrected and accelerated confidence interval based on 2,000 bootstrap samples; LL = lower limit; UL = upper limit; PFB-K = Partnerschaftsfragebogen (partnership questionnaire); F-SozU K-14 = Fragebogen zur sozialen Unterstützung (perceived social support questionnaire). Significant results are in bold.
^a^ Mean-centered.
**p* < .05. ***p* < .01. ****p* < .001.

**Table S.6**

*Hierarchical Regression Analysis of Relationship Satisfaction, Perceived Social Support, and Their Interaction for Maternal Symptoms of Anger/Hostility, Controlled for Confounders and Excluding Multivariate Outliers*

|  | *B* | *SE B* | β | BCa 95% CI | | *p* | *R^2^_adj_* | *F for* Δ*R²* |
| --- | --- | --- | --- | --- | --- | --- | --- | --- |
|  |  |  |  | LL | UL |  |  |  |
| Model 1 |  |  |  |  |  |  | .004 | 2.773* |
| Constant | 1.922 | 0.253 |  | 1.418 | 2.417 | .000 |  |  |
| Number of children | 0.345 | 0.151 | **.062** | 0.048 | 0.650 | .023 |  |  |
| During lockdown | 0.032 | 0.245 | .004 | -0.473 | 0.527 | .883 |  |  |
| During easement | 0.275 | 0.200 | .047 | -0.123 | 0.650 | .162 |  |  |
| Model 2 |  |  |  |  |  |  | .052 | 19.900*** |
| Constant | 2.108 | 0.250 |  | 1.612 | 2.579 | .000 |  |  |
| Number of children | 0.259 | 0.149 | .046 | -0.026 | 0.557 | .088 |  |  |
| During lockdown | -0.083 | 0.239 | -.012 | -0.576 | 0.393 | .719 |  |  |
| During easement | 0.190 | 0.196 | .033 | -0.206 | 0.569 | .317 |  |  |
| PFB-K^a^ | -0.125 | 0.015 | **-.222** | -0.155 | -0.094 | .000 |  |  |
| Model 3 |  |  |  |  |  |  | .075 | 23.074*** |
| Constant | 2.162 | 0.247 |  | 1.697 | 2.625 | .000 |  |  |
| Number of children | 0.209 | 0.150 | .037 | -0.078 | 0.502 | .164 |  |  |
| During lockdown | -0.066 | 0.225 | -.009 | -0.521 | 0.386 | .767 |  |  |
| During easement | 0.196 | 0.188 | .034 | -0.190 | 0.573 | .302 |  |  |
| PFB-K^a^ | -0.096 | 0.016 | **-.170** | -0.124 | -0.066 | .000 |  |  |
| F-SozU K-14^a^ | -0.659 | 0.122 | **-.160** | -0.896 | -0.419 | .000 |  |  |
| Model 4 |  |  |  |  |  |  | .074 | 19.238*** |
| Constant | 2.153 | 0.249 |  | 1.678 | 2.621 | .000 |  |  |
| Number of children | 0.212 | 0.150 | .038 | -0.072 | 0.502 | .155 |  |  |
| During lockdown | -0.070 | 0.225 | -.010 | -0.512 | 0.373 | .754 |  |  |
| During easement | 0.194 | 0.188 | .033 | -0.197 | 0.568 | .304 |  |  |
| PFB-K^a^ | -0.095 | 0.015 | **-.170** | -0.124 | -0.066 | .000 |  |  |
| F-SozU K-14^a^ | -0.651 | 0.122 | **-.158** | -0.897 | -0.403 | .000 |  |  |
| PFB-K^a^ x F-SozU K-14^a^ | 0.008 | 0.024 | .010 | -0.042 | 0.057 | .740 |  |  |

*Note.* BCa CI = bias corrected and accelerated confidence interval based on 2,000 bootstrap samples; LL = lower limit; UL = upper limit; PFB-K = Partnerschaftsfragebogen (partnership questionnaire); F-SozU K-14 = Fragebogen zur sozialen Unterstützung (perceived social support questionnaire). Significant results are in bold.
^a^ Mean-centered.
**p* < .05. ***p* < .01. ****p* < .001.

**Table S.7**

*Hierarchical Regression Analysis of Relationship Satisfaction, Perceived Social Support, and Their Interaction for Paternal Symptoms of Depression, Controlled for Confounders and Excluding Multivariate Outliers*

|  | *B* | *SE B* | β | BCa 95% CI | | *p* | *R^2^_adj_* | *F for* Δ*R²* |
| --- | --- | --- | --- | --- | --- | --- | --- | --- |
|  |  |  |  | LL | UL |  |  |  |
| Model 1 |  |  |  |  |  |  | .005 | 2.465 |
| Constant | 3.282 | 0.451 |  | 2.384 | 4.207 | .000 |  |  |
| Number of children | 0.632 | 0.258 | **.083** | 0.164 | 1.105 | .018 |  |  |
| During lockdown | 0.588 | 0.470 | .057 | -0.278 | 1.481 | .218 |  |  |
| During easement | 0.213 | 0.375 | .025 | -0.497 | 0.946 | .591 |  |  |
| Model 2 |  |  |  |  |  |  | .053 | 12.728*** |
| Constant | 3.556 | 0.437 |  | 2.704 | 4.453 | .000 |  |  |
| Number of children | 0.419 | 0.256 | .055 | -0.044 | 0.905 | .102 |  |  |
| During lockdown | 0.567 | 0.458 | .055 | -0.314 | 1.465 | .221 |  |  |
| During easement | 0.195 | 0.357 | .023 | -0.486 | 0.877 | .601 |  |  |
| PFB-K^a^ | -0.189 | 0.029 | **-.223** | -0.251 | -0.131 | .000 |  |  |
| Model 3 |  |  |  |  |  |  | .096 | 18.696*** |
| Constant | 3.716 | 0.421 |  | 2.879 | 4.549 | .000 |  |  |
| Number of children | 0.255 | 0.263 | .034 | -0.247 | 0.796 | .315 |  |  |
| During lockdown | 0.607 | 0.437 | .058 | -0.260 | 1.455 | .165 |  |  |
| During easement | 0.239 | 0.345 | .028 | -0.482 | 0.973 | .504 |  |  |
| PFB-K^a^ | -0.108 | 0.033 | **-.128** | -0.174 | -0.042 | .002 |  |  |
| F-SozU K-14^a^ | -1.239 | 0.212 | **-.232** | -1.664 | -0.837 | .000 |  |  |
| Model 4 |  |  |  |  |  |  | .098 | 16.105*** |
| Constant | 3.822 | 0.428 |  | 2.989 | 4.669 | .000 |  |  |
| Number of children | 0.267 | 0.261 | .035 | -0.225 | 0.796 | .291 |  |  |
| During lockdown | 0.538 | 0.441 | .052 | -0.331 | 1.405 | .222 |  |  |
| During easement | 0.205 | 0.347 | .024 | -0.485 | 0.919 | .568 |  |  |
| PFB-K^a^ | -0.112 | 0.033 | **-.132** | -0.178 | -0.046 | .001 |  |  |
| F-SozU K-14^a^ | -1.330 | 0.223 | **-.249** | -1.777 | -0.903 | .000 |  |  |
| PFB-K^a^ x F-SozU K-14^a^ | -0.062 | 0.036 | -.060 | -0.133 | 0.004 | .086 |  |  |

*Note.* BCa CI = bias corrected and accelerated confidence interval based on 2,000 bootstrap samples; LL = lower limit; UL = upper limit; PFB-K = Partnerschaftsfragebogen (partnership questionnaire); F-SozU K-14 = Fragebogen zur sozialen Unterstützung (perceived social support questionnaire). Significant results are in bold.
^a^ Mean-centered.
**p* < .05. ***p* < .01. ****p* < .001.

**Table S.8**

*Hierarchical Regression Analysis of Relationship Satisfaction, Perceived Social Support, and Their Interaction for Paternal Symptoms of Anger/Hostility, Controlled for Confounders and Excluding Multivariate Outliers*

|  | *B* | *SE B* | β | BCa 95% CI | | *p* | *R^2^_adj_* | *F for* Δ*R²* |
| --- | --- | --- | --- | --- | --- | --- | --- | --- |
|  |  |  |  | LL | UL |  |  |  |
| Model 1 |  |  |  |  |  |  | .010 | 3.904** |
| Constant | 1.238 | 0.268 |  | 0.734 | 1.798 | .000 |  |  |
| Number of children | 0.377 | 0.152 | **.090** | 0.105 | 0.671 | .015 |  |  |
| During lockdown | 0.461 | 0.286 | .080 | -0.096 | 1.004 | .106 |  |  |
| During easement | 0.029 | 0.216 | .006 | -0.455 | 0.452 | .891 |  |  |
| Model 2 |  |  |  |  |  |  | .039 | 9.407*** |
| Constant | 1.355 | 0.267 |  | 0.875 | 1.911 | .000 |  |  |
| Number of children | 0.286 | 0.152 | .068 | 0.012 | 0.583 | .058 |  |  |
| During lockdown | 0.452 | 0.282 | .079 | -0.095 | 0.968 | .112 |  |  |
| During easement | 0.022 | 0.214 | .005 | -0.435 | 0.426 | .912 |  |  |
| PFB-K^a^ | -0.081 | 0.016 | **-.173** | -0.114 | -0.050 | .000 |  |  |
| Model 3 |  |  |  |  |  |  | .047 | 9.259*** |
| Constant | 1.396 | 0.262 |  | 0.885 | 1.894 | .000 |  |  |
| Number of children | 0.243 | 0.155 | .058 | -0.046 | 0.553 | .121 |  |  |
| During lockdown | 0.462 | 0.271 | .081 | -0.060 | 1.000 | .089 |  |  |
| During easement | 0.033 | 0.203 | .007 | -0.401 | 0.424 | .867 |  |  |
| PFB-K^a^ | -0.060 | 0.019 | **-.129** | -0.099 | -0.022 | .001 |  |  |
| F-SozU K-14^a^ | -0.320 | 0.117 | **-.108** | -0.542 | -0.081 | .007 |  |  |
| Model 4 |  |  |  |  |  |  | .051 | 8.513*** |
| Constant | 1.471 | 0.263 |  | 0.954 | 1.976 | .000 |  |  |
| Number of children | 0.251 | 0.155 | .060 | -0.038 | 0.565 | .108 |  |  |
| During lockdown | 0.413 | 0.268 | .072 | -0.102 | 0.945 | .126 |  |  |
| During easement | 0.009 | 0.200 | .002 | -0.415 | 0.396 | .968 |  |  |
| PFB-K^a^ | -0.063 | 0.019 | **-.134** | -0.102 | -0.025 | .001 |  |  |
| F-SozU K-14^a^ | -0.385 | 0.116 | **-.130** | -0.617 | -0.152 | .001 |  |  |
| PFB-K^a^ x F-SozU K-14^a^ | -0.044 | 0.018 | **-.077** | -0.079 | -0.009 | .017 |  |  |

*Note.* BCa CI = bias corrected and accelerated confidence interval based on 2,000 bootstrap samples; LL = lower limit; UL = upper limit; PFB-K = Partnerschaftsfragebogen (partnership questionnaire); F-SozU K-14 = Fragebogen zur sozialen Unterstützung (perceived social support questionnaire). Significant results are in bold.
^a^ Mean-centered.
**p* < .05. ***p* < .01. ****p* < .001.
